# Depressed Mood Differentially Mediates the Relationship between Pain Intensity and Pain Disability Depending on Pain Duration: A Moderated Mediation Analysis in Chronic Pain Patients

**DOI:** 10.1155/2016/3204914

**Published:** 2016-05-29

**Authors:** Thomas Probst, Susanne Neumeier, Jürgen Altmeppen, Michael Angerer, Thomas Loew, Christoph Pieh

**Affiliations:** ^1^Department of Psychology, University of Regensburg, 93053 Regensburg, Germany; ^2^Department of Psychology and Psychotherapy, Witten/Herdecke University, Witten, 58448 Herdecke, Germany; ^3^Interdisciplinary Pain Clinic, Weiden Hospital, 92637 Weiden, Germany; ^4^Department of Anesthesiology, Weiden Hospital, 92637 Weiden, Germany; ^5^Department of Neurology, Weiden Hospital, 92637 Weiden, Germany; ^6^Department of Psychosomatic Medicine, University Hospital Regensburg, 93053 Regensburg, Germany; ^7^Department for Psychotherapy and Biopsychosocial Health, Danube University Krems, 3500 Krems, Austria

## Abstract

Research has shown that pain is associated with disability and that depressed mood mediates the relationship between pain and disability. The question of whether duration of pain moderates these effects was addressed in this cross-sectional study with 356 chronic pain patients. A simple mediation model replicated the notion that depressed mood explains a significant proportion of the relationship between pain and disability (in the study at hand: 12%). A moderated mediation model revealed that the indirect effect of pain on disability through depressed mood is moderated by pain duration: while depressed mood did not mediate the effect of pain on disability in chronic pain patients with shorter pain duration, depressed mood significantly mediated the effect pain exerts on disability in chronic pain patients with longer pain duration. Pain duration did not moderate the direct effect of pain on disability. Implications of these findings for the treatment of chronic pain might be that targeting depressed mood is especially relevant in chronic pain patients with longer pain duration to reduce the effect of pain on disability.

## 1. Introduction

Pain is a highly prevalent disorder [1, 2] that is frequently associated with psychiatric comorbidity [3, 4], involves enormous economic and societal costs [5, 6], and causes serious disability [7, 8]. Because the underlying mechanisms of how pain leads to disability are not completely understood, a meta-analysis was performed and found self-efficacy, psychological distress, and fear to be significant mediators of the effect of pain on disability [9]. Mediation and moderation analyses [10] are promising methods to achieve a deeper understanding of the psychopathological processes underlying the pain-disability link. While a moderator is a variable that influences the strength of the relationship between one variable *X* and another variable *Y*, a mediator is a variable that fully or partially explains the effect one variable *X* exerts on another variable *Y*. For example, mediation studies revealed that depressed mood, a frequent psychopathology among pain patients [11–13], explains a significant proportion of the effect of pain on disability in acute [14], subacute [15], and chronic pain patients with relatively short pain durations of on average 3.6 years [16]. These observations and findings of poorer treatment outcomes for pain patients with depression [13, 17] highlight the importance of preventing and treating depression in pain patients. Among the variables found to be associated with a higher risk of depression among pain patients are demographic variables such as female gender [18, 19], psychological variables (e.g., low locus of control [20]), and pain-related variables: specific pain locations, number of pain locations, and severity of pain were associated with onset of depression and anxiety [21]. Duration of pain, however, did not correlate with measures of depression in previous research [21, 22], although longer pain duration has been found to be associated with more severe disability in pain patients [23]. These controversial results underline the importance of disentangling the associations between pain, disability, depression, and pain duration. In the abovementioned meta-analysis, it was the authors' intention to address whether mediators differentially influence the pain-disability association depending on duration of pain; however, it was not possible to investigate the moderating role of pain duration due to the limited number of studies per pain duration subgroup [9]. Therefore, the question of whether pain duration impacts the direct effect of pain on disability remains as unclear as the question of whether pain duration influences the indirect effect of pain on disability through the mediator depressed mood. To fill this gap, the present study was conducted and two research questions were addressed. First, we investigated in a simple mediation model whether depressed mood mediates the effect of pain on disability in long-term chronic pain patients. Second, we extended this simple mediation model to a moderated mediation model [10] to explore the impact of the potential moderator pain duration.

## 2. Method

### 2.1. Participants

This is a retrospective analysis of patients with nonmalignant pain undergoing the pain management program at the pain clinic in Weiden, Germany [24].

### 2.2. Measures

This study included the patients with available pretreatment scores on the following measures.

*Numeric Rating Scale (NRS) [25, 26]*. The NRS is a reliable and valid 11-point numeric scale ranging from 0 (no pain intensity) to 10 (worst possible pain intensity). The NRS can be used to rate current pain intensity as well as minimum, average, or maximum pain intensity for different time intervals. In this paper, the average pain intensity rating referring to the past 4 weeks (NRS average) was used to operationalize a patient's pain intensity.

*Pain-Disability Index (PDI) [27, 28]*. The PDI is a psychometrically sound self-report to measure pain-related disability [29]. The patients are asked to rate the degree to which pain interferes with functioning from 0 (no disability) to 10 (total disability) in 7 broad areas: family/home responsibilities, recreation, social activity, occupation, sexual behavior, self-care, and life-support activity. The PDI global score was used in the current study to assess the patients' disability.

*Center for Epidemiological Studies Depression Scale (CES-D) [30, 31]*. The CES-D is a reliable and valid self-report to measure depressed mood. It comprises 20 depressive symptoms that are rated on a 4-point Likert scale ranging from 0 to 3. The global score of the CES-D was analyzed in the present study as a measure of depressed mood.

*Pain Duration*. The duration of pain was estimated in months by the patients retrospectively before the start of the treatment.

### 2.3. Source of Funding and Ethical Considerations

This study was planned and conducted in accordance with the Declaration of Helsinki and ethical laws pertaining to the medical professions. All participants signed consensus declaration and agreed to the analysis of their anonymous data. This study was conducted independent of any institutional influence and was not funded externally.

### 2.4. Statistics

The statistical analyses were performed with SPSS 23. Moreover, the SPSS macro PROCESS was used for the moderation and mediation analyses. PROCESS uses bootstrapping, a resampling technique, to obtain confidence intervals of indirect effects. The bootstrap confidence intervals offer several advantages over intervals derived from methods assuming normality of the sampling distribution (e.g., Sobel test) [10]. The study at hand estimated the confidence intervals of the indirect effect with 10.000 bootstrap samples because Hayes recommended the use of 10.000 bootstrap samples [10].

Frequencies (*n*), percentages (%), means (M), and standard deviations (SD) were calculated for the sample description.

Pearson correlation coefficients were performed to calculate bivariate correlations between pain intensity (NRS), pain disability (PDI), depressed mood (CES-D), and pain duration.

**Figure 1:**
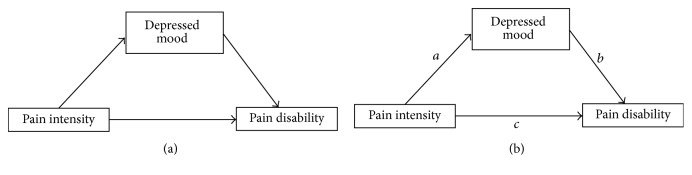
Conceptual (a) and statistical (b) diagrams of the simple mediation model [10].

For research question 1, that is, whether depressed mood mediates the effect of pain intensity on pain disability, we used PROCESS [10] and performed a simple mediation model with 10.000 bootstrap samples and applied a confidence interval of 95% (bias corrected). Pain intensity (NRS) was added as predictor, pain disability (PDI) as outcome, and depressed mood (CES-D) as mediator.  Figure 1 depicts the conceptual and statistical diagrams of this simple mediation model.

**Figure 2:**
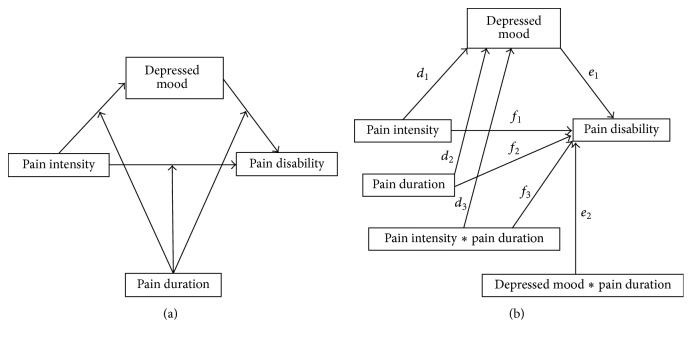
Conceptual (a) and statistical (b) diagrams of the moderated mediation model [10].

To address research question 2, that is, whether pain duration moderates the direct effect of pain intensity on pain disability and/or the indirect effect of pain intensity on pain disability through depressed mood, a moderated mediation model was performed with PROCESS [10]. Again, 10.000 bootstrap samples and a confidence interval of 95% were selected (bias corrected). Once more, pain intensity (NRS) was entered as predictor, pain disability (PDI) as outcome, and depressed mood (CES-D) as mediator. In addition, pain duration was added as a moderator.  Figure 2 illustrates the conceptual and statistical diagrams of this moderated mediation model.

All statistical tests performed were two-tailed, no statistical corrections for multiple testing were applied, and the significance value was set to *p* < .05 (95% confidence intervals). As an effect size, the ratio of the indirect effect to the total effect [10] was used because this measure was applied in prior studies on the mediating role of depressed mood [9, 14, 15].

## 3. Results

### 3.1. Sample

This study analyzed data from *N* = 356 patients with a pain duration of at least 6 months and available pretreatment data on the following variables: NRS average, PDI, CES-D, and pain duration. The characteristics of the sample are presented in Table 1.

### 3.2. Correlations

The bivariate Pearson correlation coefficients for the variables studied in this paper are presented in Table 2.

### 3.3. Mediation

The results for the simple mediation model are summarized in Table 3. It can be seen that the direct effect of pain intensity on pain disability remained significant when statistically controlling for depressed mood (*c* = 2.42; *p* < .01). However, the bias corrected bootstrap confidence intervals revealed that the indirect effect of pain intensity on pain disability through depressed mood is larger than zero (*a∗b* = .33; lower level of the confidence interval: .05; upper level of the confidence interval: .67). Depressed mood functioned, therefore, as a partial mediator in the relationship between pain intensity and pain disability. The percentage of the total effect explained by the indirect effect amounted to 12% (lower level of the confidence interval: 2%; upper level of the confidence interval: 24%).

### 3.4. Moderated Mediation

Table 4 shows the results for the moderated mediation model. While neither the interaction effect between pain duration and pain intensity on pain disability (*f*_3_ < .01; *p* = .95) nor the interaction effect between pain duration and depressed mood on pain disability (*e*_2_ < .01; *p* = .22) attained statistical significance, the interaction effect between pain duration and pain intensity on depressed mood reached statistical significance (*d*_3_ = .01; *p* = .01). The positive estimate of the interaction effect between pain intensity and pain duration on depressed mood means that increases in pain duration intensified the effect of pain intensity on depressed mood.

These results indicate that pain duration did not moderate the direct effect of pain on disability, whereas the indirect effect of pain on disability through depressed mood was moderated by pain duration. The indirect effect is conditional because it is a product of a conditional effect (effect of pain on depressed mood) and an unconditional effect (effect of depressed mood on disability) [10].

To achieve a deeper understanding of these results, the direct and indirect effects are displayed for different values (10th, 25th, 50th, 75th, and 90th percentiles) of the moderator pain duration in Table 4. While the direct effect of pain on disability was significant for very low (12 months), low (24 months), moderate (60 months), high (120 months), and very high (204 months) pain duration values, the indirect effect of pain on disability through depressed mood was above zero (referring to the bias corrected bootstrap confidence intervals) only at high (estimate for 120 months = .51; lower level of the confidence interval: .22; upper level of the confidence interval: .94) and very high (estimate for 204 months = .77; lower level of the confidence interval: .14; upper level of the confidence interval: 1.80) values of the moderator pain duration. The corresponding effect sizes (ratio of the indirect effect to the total effect) for the mediator depressed mood amounted to 17% for high (120 months) and 23% for very high (204 months) values of pain duration, whereas the effect sizes did not attain statistical significance for very low (12 months), low (24 months), and moderate (60 months) pain duration values.

## 4. Discussion

Recently, it has been stated that pain duration could be a relevant moderator of mediation effects between pain and disability [9]; its empirical impact, however, has, to our knowledge, not yet been scrutinized. Previous research that left the moderating role of pain duration out of consideration found that depressed mood explains a significant proportion of the effect of pain on disability in acute [14], subacute [15], and chronic pain patients with relatively short pain durations [16]. This result was replicated for long-term chronic pain patients in the present study by a simple mediation model that did not include pain duration as a moderator. While the chronic pain patients analyzed by Seekatz and colleagues had a mean pain duration of M = 3.6 years [16], our sample suffered on average more than twice as long from pain (M = 7.3 years). In the current study, 12% of the total effect of pain on disability occurred indirectly through depressed mood. In comparison, Hall and colleagues, for example, found that depressed mood explains 27% of the total effect between pain and disability in subacute pain patients [15]. Their estimate is close to but slightly above the upper level of our 95% confidence interval for the effect size (ratio of indirect to total effect) of the mediator depressed mood that ranged from 2% to 24%. These differences could at least be partially explained by the different samples (subacute versus chronic pain), the different designs (longitudinal versus cross-sectional), and the different measures used to operationalize depressed mood or disability. The last point is supported by findings that the effect size of the mediator depressed mood in the pain-disability relationship is more or less strong depending on the operationalization of disability [16]: depressed mood explained more of the effect of pain on psychological functioning than of the effect pain exerts on physical functioning [16]. The measure of disability in the study at hand, the Pain-Disability Index (PDI) [27], assesses pain-related disability in seven areas (family/home responsibilities, recreation, social activity, occupation, sexual behavior, self-care, and life-support activity) and future research could evaluate whether pain exerts a more or less strong effect through depressed mood on specific PDI areas.

The mediation analyses discussed until now did not take the moderating role of pain duration into account. Therefore, we aimed to elucidate the impact of pain duration in a moderated mediation model. That model explored whether pain duration moderates the direct effect of pain on disability and/or the indirect effect of pain on disability through depressed mood. Although the direct effect of pain on disability was not moderated by pain duration, the moderated mediation model revealed that the indirect effect of pain on disability through depressed mood is moderated by pain duration: depressed mood was not a significant mediator between pain and disability at very low (12 months), low (24 months), and moderate (60 months) values of pain duration; for chronic pain patients with higher (120 months) and very high (204 months) pain duration values, however, depressed mood was a significant mediator and explained a significant proportion of the total effect pain exerts on disability that amounted to 17% (for 120 months) and 23% (for 204 months), respectively. Interestingly, the moderated mediation emerged because of one specific mechanism: pain duration moderated the path from pain to depressed mood, but not the path from depressed mood to disability. Regarding the moderated path from pain to depressed mood, increases in duration of pain were associated with increases of the effect pain exerts on depressed mood. More specifically, pain was significantly associated with depressed mood only in chronic pain patients with high (120 months) and very high (204 months) values of pain duration. This result could be explained by the learned helplessness theory [32]: the longer the duration of pain is, the more likely it is to experience the pain as uncontrollable and inescapable, which in turn is a risk factor for depression [11, 33]. Due to the retrospective nature of the present study, helplessness and variables already shown to be important in the pain-disability relationship (e.g., self-efficacy and fear [9]) were not assessed and, thus, could not be explored as mediators. Therefore, future research needs to evaluate whether the effects of other mediators between pain and disability are also moderated by pain duration. Focusing solely on the one mediator depressed mood is to our view a major shortcoming of this study. A further limitation is that the results rely on a cross-sectional study that does not allow drawing causal inferences. It has been shown that pain and depression influence each other [34, 35], that depression predicts pain and disability [36], and that pain impacts subsequent depression [37]. In the study at hand, such temporal associations could not be explored due to the cross-sectional design. Another shortcoming of the current study is the heterogeneity of the sample with different pain types and regions. Future research could, hence, explore whether the moderating effect of pain duration is differential for pain types or pain regions. It could be possible that depressed mood mediates the effect of pain on disability only at higher values of pain duration for specific pain types/regions, but already at lower values of pain duration for other specific pain types/regions. For example, depressed mood has been shown to mediate the effect of pain on disability in a more homogenous sample of chronic back pain patients with pain duration values (M ± 1 SD: 24–62 months [16]) at which depressed mood was not a significant mediator in our heterogeneous chronic pain sample. Regarding duration of pain, it should be kept in mind that we evaluated subjective retrospective ratings of pain duration that could be biased. Nevertheless, the nonsignificant correlations between pain duration on the one hand and pain intensity, depressed mood, and pain disability on the other hand show that the subjective pain duration ratings were not significantly influenced by these variables. The lack of significant associations between pain duration and depressed mood in pain patients is in line with the results of other studies [21, 22]. In contrast to previous research [23], however, pain duration did not correlate with disability in the study at hand. Again, different operationalizations of disability might at least partially account for this discrepancy.

To summarize the most important results of this study, pain increases disability in chronic pain patients regardless of pain duration, whereas pain enhances disability through depressed mood only in chronic pain patients with longer pain duration. In the current psychiatric research, disentangling the pain-depression link is considered an important issue [38] and in this context our results might have the following implications for the treatment of chronic pain. First, treating pain intensity could reduce the effect of pain on disability in chronic pain patients regardless of the patients' pain duration. However, as recently discussed, targeting predominately pain intensity might be suboptimal in the treatment of chronic pain patients [39, 40]. Reducing psychopathologies such as depressed mood and developing coping or acceptance capabilities could be further treatment options in chronic pain patients [17, 41, 42], whereby, as suggested by the results of the present study, reducing depressed mood might be more essential in chronic pain patients with a long pain duration than in chronic pain patients with a short pain duration. We want to stress at this point that our analyses rely solely on the outcome disability. Therefore, pain patients with shorter pain duration might also profit from therapies targeting depression when another outcome is considered; for example, treating depression might prevent a chronic course [43].

In conclusion, our results support previous findings that depression is a mediator in the relationship between pain intensity and disability. Moreover, the result that pain duration moderates the effect pain intensity exerts on disability through depressed mood adds novelty to this fact. Our results indicate that preventing and treating depressed mood are highly relevant in chronic pain patients with longer pain durations to reduce the effect of pain on disability.

## Figures and Tables

**Table 1:** Sample description.

Variable	Statistics
Age	
M (SD) [min.; max.]	48.39 (10.31) [20; 74]

NRS average	
M (SD) [min.; max.]	6.96 (1.70) [1; 10]

PDI	
M (SD) [min.; max.]	39.17 (13.60) [0; 70]

CES-D	
M (SD) [min.; max.]	27.20 (10.74) [0; 57]

Pain duration in months	
M (SD) [min.; max.]	87.09 (79.38) [6; 432]

Number of comorbid psychiatric diagnoses	
M (SD) [min.; max.]	2.15 (1.15) [0; 7]

ICD-10 depression diagnoses (F32, F33, F34.1)	
Yes	223 (62.64)
No	133 (37.36)

Gender *N* (%)	
Female	178 (50.00)
Male	178 (50.00)

Pain chronicity stage (MPSS) *N* (%)	
1	12 (3.37)
2	106 (29.78)
3	238 (66.85)

Education *N* (%)	
<9 years	13 (3.67)
9-10 years	316 (89.27)
11–13 years	15 (4.24)
>13 years	10 (2.82)

*Note.* M: mean; SD: standard deviation; NRS: Numeric Rating Scale; PDI: Pain-Disability Index; CES-D: Center for Epidemiological Studies Depression Scale; MPSS: Mainz Pain Staging System [44].

**Table 2:** Bivariate correlations (Pearson correlation coefficients) between pain intensity, pain disability, depressed mood, and pain duration.

	Pain disability (PDI)	Depressed mood (CES-D)	Pain duration
Pain intensity (NRS average)	.34^*∗∗*^	.11^*∗*^	.04
Pain disability (PDI)	—	.41^*∗∗*^	.08
Depressed mood (CES-D)	—	—	.08

*Note.* ^*∗∗*^ *p* < .01;  ^*∗*^ *p* < .05; NRS: Numeric Rating Scale; PDI: Pain-Disability Index; CES-D: Center for Epidemiological Studies Depression Scale.

**Table 3:** Results of the simple mediation analysis investigating depressed mood as a mediator between pain intensity and pain disability.

*Normal theory test*						
	Coeff.	SE	*t*	*p*	LLCI	ULCI
Effect of pain intensity on depressed mood (*a* path)	.70	.33	2.10	.04	.04	1.36
Effect of depressed mood on pain disability (*b* path)	.47	.06	8.06	<.01	.36	.59
Direct effect of pain intensity on pain disability (*c* path)	2.42	.37	6.53	<.01	1.69	3.15

*Bootstrap results for the indirect effect*						
	Effect	Boot SE	Boot LLCI		Boot ULCI	

Indirect effect of pain intensity on pain disability through depressed mood (*a* × *b* path)	.33	.15	.05		.67	

*Note.* Coeff.: coefficient; SE: standard error; LLCI: lower level of the 95% confidence interval; ULCI: upper level of the 95% confidence interval.

**Table:** (a) Consequent

Antecedent		Depressed mood (mediator)				Pain disability (outcome)		
		Coeff.	SE	*p*		Coeff.	SE	*p*
Pain intensity (predictor)	*d*_1_	−.21	.49	.67	*f*_1_	2.43	.56	<.01
Pain duration (moderator)	*d*_2_	−.07	.03	.04	*f*_2_	.03	.04	.42
Pain intensity *∗* pain duration	*d*_3_	.01	<.01	.01	*f*_3_	<.01	.01	.95
Depressed mood (mediator)		—	—	—	*e*_1_	.55	.09	<.01
Depressed mood *∗* pain duration		—	—	—	*e*_2_	<.01	<.01	.22

**Table:** (b) Conditional direct effects of pain intensity on pain disability

Pain duration (percentiles)	Effect	SE	*t*	*p*	LLCI	ULCI
12 months	2.44	.51	4.79	<.01	1.44	3.44
24 months	2.44	.47	5.24	<.01	1.53	3.36
60 months	2.45	.38	6.43	<.01	1.70	3.20
120 months	2.47	.45	5.46	<.01	1.58	3.36
204 months	2.50	.81	3.07	<.01	.90	4.10

**Table:** (c) Conditional indirect effects of pain intensity on pain disability through depression

Pain duration (percentiles)	Effect	Boot SE	Boot LLCI	Boot ULCI	Effect size
12 months	−.04	.22	−.49	.39	−0.02
24 months	.03	.20	−.37	.43	0.01
60 months	.24	.16	−.06	.56	0.09
120 months	.51	.18	.22	.94	0.17
204 months	.77	.41	.14	1.80	0.23

*Note.* Coeff.: coefficient; SE: standard error; LLCI: lower level of the 95% confidence interval; ULCI: upper level of the 95% confidence interval; percentiles: 10th, 25th, 50th, 75th, and 90th percentiles; effect size: ratio of the indirect effect to the total effect.
